# B cells interactions in lipid immune responses: implications in atherosclerotic disease

**DOI:** 10.1186/s12944-016-0390-5

**Published:** 2017-02-06

**Authors:** Laura C Echeverri Tirado, Lina M Yassin

**Affiliations:** Facultad de Medicina, Universidad CES, Calle 10 A Nro. 22-04, Medellín, Colombia

**Keywords:** B cells, Lipids, Atherosclerosis, Cardiovascular diseases, Scavenger receptors, Fc receptors, CD1 receptors

## Abstract

Atherosclerosis is considered as an inflammatory and chronic disorder with an important immunologic component, which underlies the majority of cardiovascular diseases; condition that belongs to a group of noncommunicable diseases that to date and despite of prevention and treatment approaches, they remain as the main cause of death worldwide, with 17.5 million of deaths every year. The impact of lipids in human health and disease is taking center stage in research, due to lipotoxicity explained by elevated concentration of circulating lipids, in addition to altered adipose tissue metabolism, and aberrant intracellular signaling. Immune response and metabolic regulation are highly integrated systems and the proper function of each one is dependent on the other. B lymphocytes express a variety of receptors that can recognize foreign, endogenous or modified self-antigens, among them oxidized low density lipoproteins, which are the main antigens in atherosclerosis. Mechanisms of B cells to recognize, remove and present lipids are not completely clear. However, it has been reported that B cell can recognize/remove lipids through a range of receptors, such as LDLR, CD1d, FcR and SR, which might have an atheroprotector or proatherogenic role during the course of atherosclerotic disease. Pertinent literature related to these receptors was examined to inform the present conclusions.

## Background

Cardiovascular diseases (CVDs) belong to a group of noncommunicable diseases (NCDs) that to date and despite of prevention and treatment approaches, they remain as the main cause of death worldwide [1, 2]. Atherosclerosis is considered as an inflammatory and chronic disorder with an important immunologic component, which underlies the majority of CVDs [3–5]. This process is favored by inadequate life styles like the use of cigarette, physical inactivity, unhealthy diets, harmful use of alcohol and in some cases even genetic susceptibilities; leading to metabolic changes and promoting the development of other kind of conditions like hypertension, dyslipidemia, overweight and diabetes, which constitute comorbidities of the so called “metabolic syndrome” (MetS) [6–8].

In 1998 the first formal definition of MetS was published by the World Health Organization (WHO) which specifies that besides insulin resistance, that results to be the major underlying risk factor of the MetS, two additional risk factors are necessary for MetS diagnosis, such as obesity, hypertension, high triglyceride levels, reduced high-density lipoprotein (HDL) levels, or microalbuminuria. However, the latest reported definition, communicated by representatives of the International Diabetes Federation; National Heart, Lung and Blood Institute; American Heart Association among others in 2009, considers abdominal obesity as a mandatory requirement for the diagnosis of MetS, including the presence of any of the three risk factors among the five previously described [9–14].

MetS is about dysregulation of a wide range of parameters that easily disrupt the immunological balance, through unhealthy behavioral patterns that lead to physiopathological changes, a similar case to CVD. The impact of lipids in human health and disease is taking center stage in research [15], due to lipotoxicity explained by elevated concentration of circulating lipids in non-adipose tissue, in addition to altered adipose tissue metabolism, and aberrant intracellular signaling [16]. Indeed, unbalanced production of pro and anti-inflammatory adipokines, as well as the participation of many cells of the immune system, contribute to the development of MetS, which also constitute specific features of CVD [17].

Immune response and metabolic regulation are highly integrated systems and the proper function of each one is dependent on the other. Dysfunction of these central homeostatic mechanisms can lead to a cluster of chronic metabolic disorders, characterized by “chronic inflammation”, which drives to a sustained interaction between parenchymal and stromal cells in response to exogenous and endogenous stress, ending in tissue dysfunction and remodeling [17–19]. Among this group of disorders we can find obesity, type 2 diabetes and CVD [17, 20]. In 2014, the WHO reported that NCDs continue to be the global leading cause of death; with a 46.2% associated to CVDs, representing 17.5 million deaths every year. Diabetes caused an additional 1.5 million of deaths, and it is estimated that for 2030, close to 400 million people worldwide will be affected by this disease, with 90% of these being type 2 diabetes [2, 21, 22]. Likewise, the WHO estimates that more than one billion adults worldwide are overweight, from which 300 million are clinically obese [23]. Since their projections show that NCDs will be responsible for a significant increase in the total number of deaths in the next decade, these chronic inflammatory diseases that involve lipid metabolism dysregulation are a matter of public health concern [21].

Studies of tissue morphology and transcriptional profiling in several obesity animal models as well as in obese subjects, have shown that the development of insulin resistance is related to adipose tissue remodeling, which involves cellular infiltration of macrophages inducing inflammatory pathways and ectopic lipid accumulation [24–26]. In this sense it was demonstrated that even modest levels of overweight/obesity elicit modifications in adipose tissue immune function [27].

Recently, it has been reported that early B and T lymphocyte trafficking and infiltration, could promote initiation and perpetuation of adipose tissue inflammation [28–30]. Winer et al. reported that in diet induced obese (DIO) mice, the depletion of early B cells (which currently accumulate in this model), by anti-CD20 treatment, could protect from insulin resistance despite weight gain; while transfer of DIO-IgG exacerbated the metabolic disease. This suggests that B cells contribute to insulin resistance probably by mechanisms such as antigen presentation to T cells, secreting inflammatory cytokines and/or producing pathogenic antibodies [22, 31]. Also, in diabetes mellitus patients, alterations of B cell toll like receptor (TLR) function have been described favoring inflammation, by increased expression of surface TLR4, elevation of pro-inflammatory IL8, and decreased levels of the anti-inflammatory/protective cytokine IL10 [32]. In fact in 2002, the Leiden 85-Plus study found a direct association between the decreased IL10 levels, type 2 diabetes and MetS [33]. In parallel, B cell activation is usually detected in both inherited (e.g., Gaucher disease) [34], and acquired lipid metabolism disorders such as atherosclerosis, in which its participation is contradictory. Some results showed increased atherosclerosis in B cell deficient LDL receptor-deficient (*Ldlr* ^*−/−*^) mice, suggesting an atheroprotector role, whereas others have reported that the depletion of B cells through the use of specific monoclonal antibodies against CD20 molecule, reduce the development of atherosclerosis in both, apolipoprotein E single knockout (*Apoe* ^*−/−*^) and *Ldlr* ^*−/−*^ mice, showing an atherogenic effect [35, 36].

B lymphocytes express a variety of receptors that recognize foreign, endogenous or modified self-antigens, among them oxidized low density lipoproteins (oxLDL), which are the main antigens in atherosclerosis. B cell mechanisms to recognize, remove and present lipids are not completely clear. However, it has been reported that B cells can remove lipid antigens through a B cell receptor (BCR) dependent via, but also there is internalization and antigen presentation to invariant natural killer T (iNKT) cells by BCR independent via, associated to low density lipoprotein receptor (LDLR) expression on activated B cells [37–41].

CD1 is included among the receptors expressed in B cells that have the ability to present lipid antigens. They belong to β2 microglobulin family associated polypeptides, which relate with the major histocompatibility complex (MHC) class I and II. Also, B cells express Fc receptors (FcR), which through their immunoreceptor tyrosine-based activation or inhibitory motifs (ITAMs or ITIMs) initiate and propagate early signaling events leading to cell-specific responses [42], and scavenger receptors (SR), which belong to the family of pattern recognition receptors (PRR), that recognize pathogen associated molecular patterns (PAMPs), as well as modified antigens, such as death associated molecular patterns (DAMPs), including modified host derived molecules like oxLDL [43, 44] (Table 1).

**Table 1:** Receptors involved in lipid recognition-removal or presentation and immune responses in experimental atherosclerotic disease

	Receptor	Described Mechanism	Receptor Subclass	Results in experimental animal model	Reported Role in atherosclerosis	Ref.
Receptors involved in lipids recognition-removal or presentation by B cells	LDLR	Internalization of LDL through LDLR, to transfer cholesterol from plasma LDL into the cell in a controlled manner.		A defect in the expression or internalization of LDLR leads to an increase in circulating plasma LDL, predisposing them to oxidation, condition that contributes in a great manner to the physiopathology of atherosclerosis.	Undetermined	[54, 55]
	FcR	Recognition of immunoglobulins directed to modified lipids, more specifically neoepitopes formed by lipid peroxidation.	FcμR	High titers of anti-oxLDL IgM in *Apoe* ^*−/−*^ mice fed a high fat diet.	Undetermined	[58, 59]
			FcγRIIB	Deficiency in the γ chain expression of FcγR in *Apoe* ^*−/−*^mice fed with high fat diet, is related with a limited development and progression of atherosclerosis that could be associated with the loss of FcγRI and FcγRIIIA, and the overexpression of the inhibitory FcγRIIB characteristic of this mouse model.	Atheroprotector	[69, 70]
				Absence of FcγRIIB induces increased atherosclerosis development in *Ldlr* ^*−/−*^atherosclerosis mouse model, as well as increased activation and expansion of B cells.	Undetermined	[71]
				The absence of FcγRIIB induces increased atherosclerosis development in *Ldlr* ^*−/−*^atherosclerosis mouse model, as well as increased activation and expansion of B cells.		[72]
	SR	Recognition of several ligands, such as microbial, environmental, endogenous and self-modified antigens, either by endocytosis or phagocytosis, contributing to the outcome of the immune system responses.	CD36	The absence of CD36 protects against atherosclerotic lesion development in *Apoe* ^*−/−*^ mice, which approximately developed just a 20% of lesion assessed by *en face* analysis of whole aortas, compared with control mice.	Proatherogenic	[85–87]
				Triple knockout *CD36* ^*−/−*^ *Msr1* ^*−/−*^ *Apoe* ^*−/−*^ mice, exhibited increased serum cholesterol levels and larger atherosclerotic lesions located in aortic sinus compared with *Apoe* ^*−/−*^ mice as controls, suggesting that SR mediated lipid uptake protected against atherosclerosis lesion formation rather than promote it.	Atheroprotector	[88]
				Aortic lesion analysis in *Ldlr* ^*−/−*^ *CD36* ^*−/−*^ mice fed with western diet and *Ldlr* ^*−/−*^ mice revealed no difference between the groups, however bone marrow transplant from *Ldlr-CD36* ^*−/−*^into *Apoe* ^*−/−*^ mice had 38.4% less lesion area compared with those receiving *Ldlr* ^*−/−*^ transplant.	Proatherogenic	[89]
			SR-BI	*SRBI* ^*−/−*^ *Apoe* ^*−/−*^ mice fed with standard chow diet developed occlusive coronary artery atherosclerosis as well as significant atherosclerotic lesions, compared with control mice.	Atheroprotector	[95–97]
				Transplantation of bone marrow from *SR-BI* ^*−/−*^ mice into *Ldlr* ^*−/−*^mice, induced a twofold reduction of the mean atherosclerotic lesion area after 4 weeks of high fat diet.	Proatherogenic	[98]
	CD1	Presentation of lipid antigens or hydrophobic peptide antigens to T cells, activating a specialized T cell subset called invariant NKT cells (iNKT), and leading to immune responses that contribute with the inflammatory process.	CD1d	*CD1d* ^*−/−*^ *Ldlr* ^*−/−*^ mice present a 50% reduction in lesion formation compared with controls, but the influence on lesion progression is just transient and does not significantly affect the inflammatory cytokine milieu of mature lesions.	Proatherogenic	[118–120]

The disruption of cellular homeostasis through oxidative stress and contribution to cell death by generation of toxic intermediates during aberrant lipid metabolism, and enhanced pro-inflammatory immunological pathways, could occur in vascular, cardiac and adipose tissue diseases. This opens up the possibility that immune cells often interacting with oxidized products, and more specifically B cells, could participate in the process of lipotoxicity at the injured areas during the tissue remodeling process, leading to development and establishment of the disease or even regulating the inflammatory process. This supports the importance of understanding the receptors that could be involved in lipids recognition and/or removal by B cells.

## Receptors involved in lipids recognition-removal by B cells

### LDLR

Lymphocytes obtain cholesterol from serum low density lipoproteins (LDL) through its specific LDLR, which is internalized along with LDL; since these cells do not synthetize enough cholesterol to support their membranes [45–47]. The main function of this mechanism is to transfer cholesterol from plasma LDL into the cell in a controlled manner [48]. LDL endocytosis and subsequent lysosomal degradation induces the release of free cholesterol, which suppresses 3-hydroxy-3-methyl-glutaryl-CoA reductase (HMG-CoA) activity, stimulates acyl-CoA cholesterol acyltransferase (ACAT) and regulates the LDLR activity by a feedback mechanism [49, 50].

LDLR is widely expressed in different cell types, including T and B lymphocytes [51]. However there are important differences in cholesterol metabolism among these lymphocyte subpopulations; for example the content of both, free and ester cholesterol is slightly lower in B cells than in T cells. In fact, a conclusion of a study more than two decades ago, pointed out that B cells had deficient LDL catabolism compared with T cells, in terms of internalization of LDLR-LDL complex [52]. However, recently it has been reported that B lymphocytes purified from peripheral blood express LDLR, and are able to internalize LDL with a four-fold increase in the expression of this receptor compared with non-stimulated T and NKT cells. Also, an up regulation of this receptor has been reported after B cell activation through stimulation with different concentrations of IL2 or pokeweed mitogen, which could suggest that LDL internalization could be important for B cells metabolism and maybe even immunoglobulin (Ig) production [51]. Additionally, besides the cell type, some hormones may play a role in the regulation of LDLR activity, such as insulin, which decreases LDL metabolism in lymphocytes by lowering its binding to LDL receptor, with a consequent decreased internalization and degradation of LDL [48]. This is evident in secondary hypercholesterolemias of endocrine origin, and in diabetes, in which the lack of insulin increases the levels of triglycerides, very low density lipoproteins (VLDL) and LDL [53].

It is clear that a defect in the expression or internalization of LDLR leads to an increase in circulating plasma LDL, predisposing it to oxidation, condition that contributes in a great manner to the physiopathology of atherosclerosis [54]. Finally, it is also important to highlight that LDL removal through LDLR might be relevant not only for B cell metabolism, but also for LDL antigen presentation, which could participate in the inflammatory process observed in atherosclerosis [55].

### FcR

A critical role for FcR has been described in B cell survival following antigen presentation [56], and in an enhanced antibody response to antigenic challenge [57]. Although FcRs are not directly involved in lipids removal, they could indirectly participate in this process, due to their capability to recognize Ig directed to modified lipids. In this sense, immunological system has the ability to recognize and respond effectively to neoepitopes formed by lipid peroxidation [58]. This includes natural antibodies, which bind to oxidized phospholipids contained in oxLDL. The importance of these antibodies in the context of diseases with a lipid component is evidenced by the observation that in the *Apoe* ^*−/−*^ atherosclerosis model, mice fed with high fat diet (HFD) present high titers of anti-oxLDL IgM [59].

However, FcμR is not the only FcR expressed on B cells; actually the most widely expressed is the FcγRIIB. FcγR, are important cell surface effector molecules that bind the Fc portion of IgG. In mice, four classes of FcγRs have been identified, FcγRI, FcγRIIB, FcγRIII and FcγRIV, and three of them may interact with immune complexes, such as FcγRI, FcγRIII, and FcγRIIB [60]. This last molecule contains an ITIM in its cytoplasmic tail, leading to negative regulation of BCR signaling affecting B cell activation and differentiation [61–63].

In humans, FcγRIIA and FcγRIIB conform the FcγRII subfamily, and in both cases they bind the Fc portion of IgG with low affinity [64]. FcγRs are differentially expressed in human leukocytes. Both isoforms of CD32, respectively the activating and inhibitory members are expressed in B cells, dependent on its activation status [65]. FcγRI (CD64) and FcγRIIA (CD32) are highly expressed on monocytes, macrophages, and granulocytes, whereas FcγRIIIA (CD16) mainly in macrophages and a small subset of monocytes, as well as in smooth muscle cells and endothelial cells, which also express FcγRIIA [66], and finally FcγRIIIB which is expressed on granulocytes [67, 68].

*Apoe* ^*−/−*^mice with a deficiency in the γ chain expression of FcγR, and fed with high fat diet, exhibited a limited development and progression of atherosclerosis, suggesting a proatherogenic effect that could be associated with the loss of FcγRI and FcγRIIIA, and the overexpression of the inhibitory FcγRIIB characteristic of this mouse model. These mice also showed a ∼ 50% less atherosclerotic lesion as well as reduced expression of inflammatory molecules, such as MCP-1 and RANTES and influence over the macrophage phenotypic balance [69, 70]. This agrees with the observation that the absence of FcγRIIB induces increased atherosclerosis development in *Ldlr* ^*−/−*^ atherosclerosis mouse model, as well as increased activation and expansion of B cells [71]. However, the role of this receptor is not yet clear, since recently it was reported that FcγRIIB deficiency reduces atherosclerosis in hyperlipidemic *Apoe* ^*−/−*^ mice, as well as induction of T regulatory cells and increased secretion of atheroprotective cytokines such as IL10 and TGFβ, which are partially responsible for the attenuation of the disease [72].

Even though the role of FcR in lipid removal is not clear, their contribution in atherosclerosis development has been previously described either directly in lipids clearance or indirectly during inflammation. The role of FcγRIIB during atherosclerosis is at least partially explained by the recognition of IgG antibodies directed to oxLDL antigens, which could result in protective immunity or down regulation of pre-existing proatherogenic immune responses [71]. This is supported by the observation that human atherosclerosis lesions as well as sera from these patients, presents IgG deposits directed to oxLDL [73, 74]. The inhibitory character of FcγRIIB confers the ability to modulate B cell activity through regulation of BCR-mediated signaling, controlling proliferation, class switching, and B cell differentiation to plasma cells [75, 76], probably regulating the inflammation observed in atherosclerosis. However, activating FcγR are also involved in atherosclerosis, including FcγRIA, FcγRIIA, and FcγRIIIA, which have been described in aorta lesions from patients with atherosclerosis [66, 68].

### SR

SRs are distinguished by their wide ability to recognize several ligands, such as microbial, environmental, endogenous and self-modified antigens, either by endocytosis or phagocytosis, contributing to the outcome of the immune system responses. According to their multidomain structure, SR are classified in eight different classes: A, B, C, D, E, F, G and H [77]. In this review, we mainly focus on CD36 and SR class B type I (SR-BI), due to their proved expression on B cells and their relevance in lipid removal involved in atherosclerosis. These receptors belong to class B and D, respectively [43].

Besides macrophages and dendritic cells, other cell types including B, and some epithelial and endothelial cells express SR [78]. Specifically in B cells, a differential expression of some SRs such as CD36 and CD68 has been described, depending on the B cell subset studied. According to the differential analysis of gene expression by DNA microarrays, these receptors are expressed on marginal zone (MZ) but not on follicular (FO) B cells [79], even though for example the function of CD68 on MZ B cells remains to be determined.

### CD36

CD36 belongs to the class B SR [80], and is a type III receptor with two transmembrane domains, an extracellular loop with multiple glycosylation sites and two short intracellular tails [43]. CD36 in both murine and human versions, mediates not just the specific uptake of oxLDL, but also its intracellular accumulation and degradation, according to experiments with transfected cells with this molecule [80, 81]. This is also supported by the observation that CD36 null mice present a fast and significant increase in the levels of circulating cholesterol, non-esterified free fatty acids and triacylglycerol [82].

Although, the role of CD36 in B cells is not yet clear, it has been reported to be a specific marker for MZ B cells, due to the absence in its expression on B1 B cells. Also, it was reported that its expression could be rapidly induced on mature B cells, like FO B cells by TLR and CD40 stimulation. However, the lack of CD36 in *CD36* ^*−/−*^ mice reveal a minimal effect on mature B cells development, which is evidenced in the transitional B cells stages [83]. Major effect is observed in plasma cell generation and specific antibody responses to infectious diseases in vivo [83, 84]. Just like CD68, differential expression of CD36 in B cells, could be related with the diverse roles of B cells subsets in immune responses mediated by SRs.

In the context of lipid related diseases, it was shown that the absence of CD36 protects against atherosclerotic lesion development in *Apoe* ^*−/−*^ mice, which approximately developed just a 20% of lesion assessed by *en face* analysis of whole aortas, compared with control mice [85–87]. Whereas groups of triple knockout *CD36* ^*−/−*^ *Msr1* ^*−/−*^ *Apoe* ^*−/−*^ mice, deficient not just for CD36, but also for SR-AI and SR-AII receptors, which also recognize modified LDL such as acetylated LDL (AcLDL) and oxLDL, exhibited increased serum cholesterol levels and larger atherosclerotic lesions located in aortic sinus compared with *Apoe* ^*−/−*^ mice as controls. This suggests that the lipid uptake mediated through SR, protected against atherosclerosis lesion formation rather than promote it [88]. Finally, no difference was observed between the size of aortic lesion in *Ldlr* ^*−/−*^ *CD36* ^*−/−*^ and *Ldlr* ^*−/−*^ *CD36* ^*+/+*^ mice fed with western diet. However, bone marrow transplant from *Ldlr* ^*−/−*^ *CD36* ^*−/−*^ into *Apoe* ^*−/−*^ mice induced 38.4% less lesion area compared with those receiving *Ldlr* ^*−/−*^ *CD36* ^*+/+*^ transplant [89]. These contradictory results could indicate that the role of this receptor is not only related with aberrant lipid metabolism, but also with persistent inflammatory milieu probably modulating disease progression.

### SR-BI

SR-BI, belongs to the class B receptors of SR family, which have a loop structure similar to CD36. This receptor contains two alternative splice variants (SR-BI and SR-BII), and contrary to CD36 receptor, SR-BI recognizes mainly HDL and is responsible for reverse cholesterol transport; however it also recognizes modified lipids, among them oxLDL, as well as native LDL, suggesting that SR-BI could play a role in normal LDL metabolism [90] and could present a selective lipid uptake from HDL [91, 92].

SR-BI is expressed in different tissues such as adrenal glands, steroidogenic tissue and hepatocytes, as well as in different types of cells, like monocytes, macrophages and dendritic cells [93]. According to experiments evaluating total spleen RNA derived from *C57BL/6* mice, SR-BI as well as its splice variants, CD36 and macrophage receptor with collagenous structure (MARCO) are expressed in these cells [94]. Another observation that suggests a possible role of SR-BI in B cells, is that the dual engagement of SR-BI and TLR9 by CpG down regulates IL6 and IL10 cytokines, as well as IgM production [94].

Previously, it has been reported that SR-BI expression protects against early onset atherosclerosis development in mouse models of the disease such as *Apoe* ^*−/−*^ and *Ldlr* ^*−/−*^ [95, 96]. *SR-BI* ^*−/−*^ *Apoe* ^*−/−*^ mice fed with standard chow diet developed occlusive coronary artery atherosclerosis, as well as significant atherosclerotic lesions, compared with control mice [97]. It has furthermore been demonstrated that transplantation of bone marrow from *SR-BI* ^*−/−*^ mice into female *Ldlr* ^*−/−*^ mice, induced a twofold reduction of the mean atherosclerotic lesion area after 4 weeks of HFD. According to previous reports, SR-BI in bone marrow derived cells has a dual role in atherosclerotic lesion development, depending on disease stage [98]. Recent data from our collaborators showed that mice with advanced atherosclerosis have a variety of alterations in the frequency and phenotype of B lymphocytes, most of which were associated with dyslipidemia. This was observed in spleen and aortic tissue, suggesting the role of B cells in atherosclerosis both as a systemic and a localized disease [99, 100].

## B cells involved in lipid immune responses through CD1 receptors

### CD1

Even though CD1 is not directly involved in lipids recognition/removal, it participates in the antigen presentation of lipids, leading to immune responses that contribute with the inflammatory process. This receptors belong to a family of β2 microglobulin associated proteins related to the MHC class I and II, but encoded by genes outside the MHC. Humans have five CD1 isoforms which divide in three groups; the first one comprises CD1a, CD1b and CD1c; the second includes CD1d, and the third one CD1e molecule. In mice, there is a single class of CD1 molecule (mCD1d) which is homologous to human CD1d [101–105].

### CD1d

CD1d expression has been described in different cell types, including B cells, which express low levels of this marker [106]. In contrast to classical peptide antigen presentation, CD1 molecules have evolved to present lipid antigens or hydrophobic peptide antigens [107, 108] to T cells, activating a specialized T cell subset called iNKT cells, which express the T cell receptor (TCR) rearrangement Vα14-Jα281 (also known as Jα18 or Jα15) in mice and Vα24-Jα18 in humans [55, 109]. These TCR molecules recognize exogenous glycosphingolipid antigens such as α-galactosylceramide (α-GalCer, isolated from marine sponges), α-glucosylceramides, or diacylglycerol (isolated from the Gram-negative bacteria *Sphingomonas* and *Borrelia burgdorferi* spirochete, respectively) [39, 110–115].

In human cells, it has been reported that the interaction between iNKT and B cells trough CD1d even in the absence of α-GalCer, promote proliferation of memory and naive B lymphocytes, as well as Ig production. One of the hypothesis to explain this, suggests that B cells express an endogenous glycolipid different to α-GalCer, associated to CD1d and recognized by iNKT cells [41, 111]. Interestingly, Kain et al. have showed the presence of α-linked contaminants by blocking its stimulatory activity using antagonists, among them anti-CD1-α-GalCer antibody L363. Also confirming that β-glucosylceramides have no stimulatory properties towards NKT cells [116]. However, more recent studies conclude that β-glucosylceramides primarily constitute an NKT type II (CD1d-restricted T cells lacking iTCR) ligand, which display a distinct cytokine profile and provide robust help to B cells [34]. On the other hand, interaction of B cells with lipids is also evidenced by the observation that lipid activated iNKT cells lead to the activation of B cells. Also, it has been suggested that iNKT cells can convert tolerogenic B cells into immunogenic antigen presenting cells (APCs), which can generate long-lasting cytotoxic immunity; mechanism that could depend on the expression of functional CD1d [117]. Direct interaction of B and iNKT cells induce antibody responses during humoral immunity, which is an important mechanism dependent on the expression of CD1d on B cells [38]. This interaction could participate in the inflammatory responses that involve lipids removal and subsequent inflammation, like the one observed in atherosclerosis. In fact, it has been reported that the activation of CD1d-restricted iNKT cells exacerbates atherosclerosis [118, 119]. Additionally, the relevance of CD1d-restricted NKT cells in atherosclerosis is demonstrated by a 40% reduction in fatty streaks formation in *CD1d* ^*−/−*^ *Ldlr* ^*−/−*^ mice compared with competent CD1d *Ldlr* ^*−/−*^ mice. However, the importance of CD1d on lesion progression is just transitory and does not significantly affect the inflammatory cytokine milieu of mature lesions [120].

Also, in other chronic inflammatory disorders such as autoimmune diseases, recent data indicates that CD1d deficiency worsens autoantibody production and nephritis in a genetically susceptible lupus mouse model (BWF1) [121], as well in the hydrocarbon oil-induced model of lupus nephritis [122]. In parallel, iNKT cells participate in the regulation of autoantibody production, since autoreactive B cells are selectively reduced in the presence of activated iNKTs in a CD1d-contact dependent manner [123]. Most recently, it has also been established that inappropriate presentation of CD1d-restricted self-lipids by autoimmune B cells is a crucial mechanism leading to iNKT cell hyperactivation, proliferation, and apoptosis in autoimmune mice [124]. In the same way it has been proposed that healthy B cells are pivotal for iNKT cell homeostasis, related to the fact that in healthy donors, B cells are essential for iNKT cells expansion and activation, conditions that fail in patients with systemic lupus erythematous (SLE) due to altered CD1d recycling [125].

## Conlusions

Lipid dysregulation is the result of several inadequate life styles that progress to metabolic changes. This has huge impact during development and establishment of lipid related diseases, especially in atherosclerosis, which underlies the majority of CVDs. In this sense, immune responses and metabolic regulation are highly integrated, even a slight disruption of this interaction could lead to a silent but dangerous systemic imbalance. This is reflected by an elevated concentration of circulating lipids, due to altered metabolism of lipids, aberrant intracellular signaling, as well as unbalanced production of pro and anti-inflammatory cytokines and the participation of various immune cells. This miscommunication can transform into a steady state of inflammation, which could lead to tissue dysfunction and remodeling.

B cells might play specific roles in chronic inflammatory disorders; however it has been hard to establish because of its variability in terms of frequency and functionality among disease models, animal strains and diets. Despite the fact that other cells such as macrophages are the main subject of study in this area, participation of B cells in lipid inflammatory conditions is evidenced by the expression of a variety of receptors that can recognize foreign, endogenous or self-modified antigens such as oxidized lipids, which suggests the participation of B cells in lipotoxicity.

**Fig. 1:**
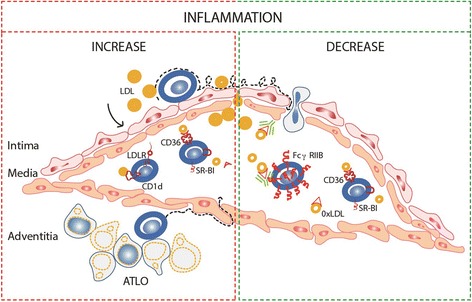
Participation of B cell receptors during atherosclerotic plaque formation. During atherosclerotic plaque formation we speculate that B cells have an important participation. The expression of LDLR contributes with the recognition of LDL molecules and its antigenic presentation trough CD1d to iNKTs exacerbates the disease. Persistence presence of LDL molecule favors its oxidation; oxLDLs are recognized by CD36 and SR-BI, leading to the development of lipid load cells either contributing to atherosclerosis (panel in red) or depending on the disease stage, diminishing the inflammatory process (panel in green). On the contrary, the up regulation of FcγRIIB receptors with inhibitory activity induce anti-inflammatory responses after recognition of immune complexes of IgG and oxLDL associated with an atheroprotector role (panel in green)

In the early stages of CVDs, hypercholesterolemia induces the diffusion of LDL particles through the activated endothelium, which in turn cause the expression of adhesion molecules which recruit, arrest and finally ends in the entrance of immune system cells to the sub-endothelial space. In mice, it has been reported that cells and soluble factors circulate from aortic tertiary lymphoid organs (ATLOs), which locate in the adventitia of diseased vessels. During the development of atherosclerotic disease, we speculate that B cells have an important participation, since the expression of LDLR contributes with the recognition of LDL molecules and its antigenic presentation trough CD1d to iNKTs exacerbates the disease. The progressive establishment of the inflammatory process and the presence of these molecules in the sub-endothelial space, induce its oxidation generating oxLDL, which are recognized by SR such as CD36, inducing an increased removal of these molecules. In parallel, we can find the up regulation of FcγRIIB receptor that through its inhibitory activity induces anti-inflammatory responses after the interaction with immune complexes of IgG and oxLDL, decreasing inflammation (Fig. 1).

All these observations open a number of possibilities regarding to the clinical application of B cells for both the early diagnosis and treatment of atherosclerosis and other lipid metabolism diseases in humans. B cells are clearly involved in lipid mediated diseases as well as in chronic inflammatory conditions, probably by the recognition of lipids through some of the receptors mentioned here, but more work needs to be done in this field.

## Abbreviations

ACAT: Acyl-CoA: cholesterol acyltransferase
AcLDL: Acetylated LDL
APCs: Antigen presenting cells
*Apoe*^*−/−*^: Apolipoprotein E single knockout
ATLOS: Aortic tertiary lymphoid organs
BCR: B cell receptor
CVDs: Cardiovascular diseases
DAMPs: Death associated molecular patterns
DIO: Diet induced obese
FcR: Fc receptors
FO: Follicular
HDL: High-density lipoprotein
HFD: High fat diet
HMG-CoA: 3-hydroxy-3-methyl-glutaryl-CoA
Ig: Immunoglobulin
iNKT: invariant Natural killer T
ITAM: Immunoreceptor tyrosine-based activation motif
ITIM: Immunoreceptor tyrosine-based inhibitory motif
LDL: Low density lipoprotein
LDLR: Low density lipoprotein receptor
*Ldlr*^*−/−*^: Low density lipoprotein receptor-deficient
MARCO: Macrophage receptor with collagenous structure
MetS: Metabolic syndrome
MHC: Major histocompatibility complex
MZ: Marginal zone
NCDs: Noncommunicable diseases
oxLDL: Oxidized low density lipoproteins
PAMPs: Pathogen associated molecular patterns
PRR: Pattern recognition receptors
SLE: Systemic lupus erythematous
SR: Scavenger receptors
SR-BI: SR class B type I
TCR: T cell receptor
TLR: Toll like receptor
VLDL: Very low density lipoproteins
WHO: World Health Organization
α-GalCer: α-galactosylceramide
